# A Privacy-Preserving Key Management Scheme with Support for Sybil Attack Detection in VANETs

**DOI:** 10.3390/s21041063

**Published:** 2021-02-04

**Authors:** L. Ellen Funderburg, Im-Yeong Lee

**Affiliations:** Department of Software Convergence, Soonchunhyang University, Asan 31538, Korea; lefunderburg@sch.ac.kr

**Keywords:** VANET, group signature, key management, Sybil attack, privacy, backward secrecy

## Abstract

Vehicular ad hoc networks (VANETs) face two important and conflicting challenges with regards to security: preserve the privacy of vehicles in order to prevent malicious entities from tracking users and detect and remove bad actors that attempt to game the system for their own advantage. In particular, detecting Sybil attacks, in which one node attempts to appear as many, seemingly conflicts with the goal of privacy preservation, and existing schemes fail on either one or both accounts. To fill this gap, we present a hierarchical key management system which uses short group signatures to preserve member privacy at lower levels while allowing mid-level nodes to detect Sybil attacks and highly trusted nodes at the top of the hierarchy to completely reveal the real identities of malicious nodes in order to prevent them from rejoining the system and for use by legal authorities. In addition, we present an argument for relaxing the requirement of backward secrecy in VANET groups in the case when no malicious activity has been detected.

## 1. Introduction

As the use of the Internet of Things (IoT) has spread from consumer devices into critical infrastructure, it is becoming more and more important to provide secure and tamper-resistant methods of IoT communication. One particular area of concern is smart transportation and autonomous vehicles. These systems combine the potential for serious physical damage with openness to connection by the general public. In such systems, it is critical to identify and remove malicious actors. On the other hand, these requirements are often in opposition with the important goal of protecting driver privacy. One example of this tension is the case of a Sybil attack, where one attacker masquerades as multiple vehicles. This paper presents a scheme that protects vehicle privacy without also preventing the detection of Sybil attacks.

Vehicular Ad hoc Networks (VANETs) are an application of Mobile Ad hoc Networks (MANETs) to vehicular environments [1]. Compared to other applications of MANETs, VANETs both move and change at faster rates [2]. Applications of VANETs include information services for human drivers, enhanced safety through driver-assistance systems, and fully autonomous vehicles.

A typical VANET system consists of VANET-equipped vehicles containing On-Board Units (OBUs) that are used for network connection and messaging, a second layer of Road Side Units (RSUs), which are specialized hardware placed next to roadways for communication with the OBUs, and a third layer of servers that are connected to the RSUs and may provide some service or data collection and monitoring for the OBUs [3].

**Figure 1:**
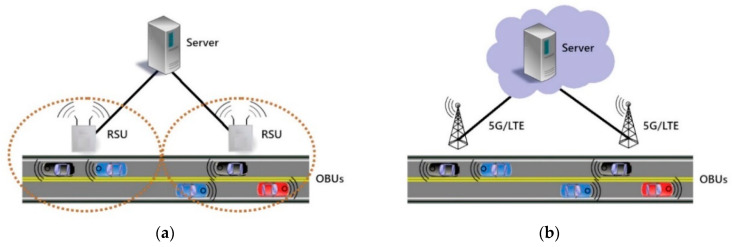
Examples of VANET configurations: (**a**) VANET with RSUs; (**b**) VANET with cloud.

The function of the RSUs varies widely with some systems using them purely as messaging relays [4], as shown in Figure 1a, while in others they are a powerful distributed layer performing some of the tasks that in other systems are reserved for the more centralized server layer [5]. With modern technology, it is possible to remove the RSUs and connect the OBUs to the rest of the system over the internet using 5G or existing cellular networks [6], which is shown by the system in Figure 1b.

**Figure 2:**
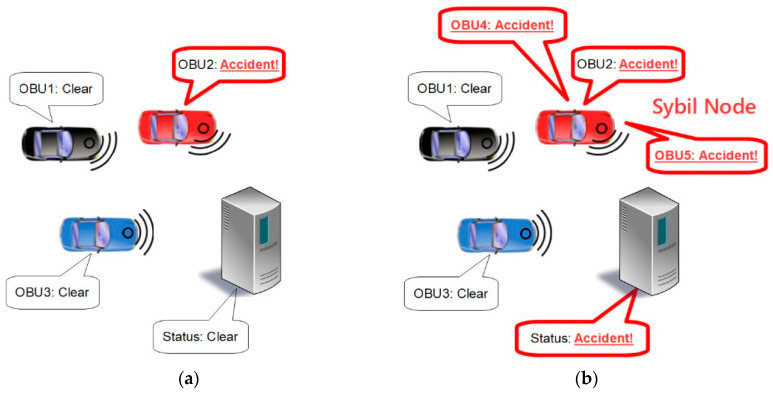
Example of greedy driver attack: (**a**) Failed attempt using a single vote; (**b**) Successful attempt using Sybil attack to create additional votes.

In VANETs, the group members exchange information about their own statuses as well as other information about the current environment and traffic patterns. This makes VANETs particularly vulnerable to a type of attack known as a Sybil attack. In a Sybil attack, one OBU would send more status messages than it should in order to create the appearance that it is actually multiple OBUs. This can create the illusion of additional traffic or it can be used to “out-vote” honest OBUs in order to deceive the system about current conditions [7]. For example, in a greedy driver attack, the OBU can report an accident where none has occurred, as shown in Figure 2, to create the appearance of congestion and cause other cars to be rerouted, leaving the greedy driver with a clear highway for itself [8].

In this paper, we address the conflicting VANET requirements of preserving the privacy of honest vehicles while still providing means to identify Sybil attacks and remove the offending vehicles from the system. In addition, our scheme will allow the conditional discovery of the true identities of malicious vehicles, which can be used to prevent them from rejoining the system and to report them to legal authorities.

The remainder of this paper is organized as follows. Section 2 presents the design background and goals. Section 3 discusses existing schemes with respect to the requirements of VANETs. Section 4 contains the details of the proposed scheme. Section 5 presents an analysis of the scheme in comparison to other schemes and in light of VANET security requirements. Section 6 concludes the paper.

## 2. VANET Security Concerns

While VANETs share many of the security concerns of other IoT systems, the high speed and frequency of connects and disconnects, as well as the publicly available nature of much of the input data, give them some unique considerations not found in many IoT systems. For example, unlike many IoT systems, confidentiality is not as important for VANETs [9]. The information transmitted in VANET messages is data that could be easily obtained by an adversary via simple observation of traffic in the area. However, authentication [10] and privacy [6] are two pressing issues. These issues are discussed in more detail below.

### 2.1. Authentication

In order to prevent malicious users from joining the system and injecting harmful data, it is important that only validated users be admitted into the system. In addition, malicious users must be prevented from rejoining the system after their detection and removal. Requiring proof and verification of real identities is one way to ensure this.

### 2.2. Privacy

The locations and identities of the vehicles in a VANET reveal a great deal of information about the habits of drivers, and the tracking of individual drivers by malicious entities can have serious consequences for those drivers. For example, criminals could learn information about a driver’s movements and use it to target them. Accordingly, the preservation of privacy is extremely important in VANET systems. The real identities of the drivers must be concealed from internal as well as external entities. Additionally, the persistent tracing of drivers by pseudo identities must also be prevented because it has been shown that even anonymized location data can be used to unmask real world identities [11].

### 2.3. Tracing

Here, tracing refers to the ability to trace malicious data back to its originator and reveal the real identity of that entity. The real identity can be used to prevent the malicious entity from rejoining the system following removal, as well as reporting that entity to the appropriate authorities.

### 2.4. Sybil Resistance

In a Sybil attack, attackers send messages that appear to come from more than one entity in order to inject misinformation into the system and perform actions such as rigging voting-based processes. For example, a malicious vehicle could send accident reports appearing to come from a large number of vehicles. If this number exceeds the number of honest vehicles reporting the true state, the system could decide that there is an accident where none exists and re-route traffic unnecessarily. It could be difficult or impossible for systems that preserve the absolute privacy of all members to detect such attacks.

### 2.5. Group Security

In addition to the above requirements, schemes which manage groups of entities, such as the proposed scheme, must also concern themselves with the entries and exits of group members. The problems considered here are forward secrecy, backward secrecy, and member revocation.

#### 2.5.1. Forward Secrecy

If a system has forward secrecy, entities joining the group cannot use their keys to read messages sent before they were members of the group [12].

#### 2.5.2. Backward Secrecy

If a system has backward secrecy, entities leaving the group can no longer use their keys to read new group messages [12]. The concept can be extended to include the requirement that group members can no longer use their keys generate valid signatures for the group.

#### 2.5.3. Revocation

Systems must have a way of removing access to the group from members who either leave the group under normal circumstances or are actively removed from the group. Once identified, it must be possible to swiftly and completely remove malicious nodes’ access to the group.

## 3. Related Work

In the last decade, there has been much work done on developing schemes for providing secure communication with VANETs. This section will briefly discuss papers covering a range of proposed VANET authentication schemes with an emphasis on their ability to facilitate Sybil attack detection while preserving the privacy of the system participants. The schemes included have been grouped according to the categories suggested in [13].

### 3.1. Protocols That Use ID-Based Signatures and Group Signatures for Authentication of Messages

A scheme using ID-based group signatures was proposed by Gao & Qi [14]. In this scheme each OBU is assigned a base pseudo-ID by the TA, which the OBU then sends to the RSU in order to obtain a list of further-anonymized pseudo-IDs to use when contacting other OBUs for vehicle-to-vehicle authentication. While the authentication is performed using group signatures, communication post-authentication takes place using 1:1 symmetric keys exchanged between the vehicles. The pre-computed list of pseudo-IDs combined with the need to store a key for every other OBU in the system creates an unnecessary overhead on storage, while also creating unnecessary communication overhead by preventing broadcast messaging. In addition, while 1:1 keys might aid in identifying malicious nodes, there’s no discussion of how the real ID identities of offending nodes might be recovered from the lists of pseudo-IDs, which is critical to detecting and recovering from Sybil attacks.

### 3.2. Protocols That Use RSUs for Authentication and/or Key Distribution

An early attempt at preserving vehicle privacy while detecting Sybil attacks was proposed by Zhou et al. [7]. The Zhou scheme uses pseudonyms that hash to identical values when using secret keys known only to the RSUs and the CA to detect Sybil attacks. The hash algorithm is efficient, but unfortunately the scheme does not provide authentication that the pseudo-IDs used in the messages were legitimately assigned to the vehicles using them. Additionally, there is no explicit mechanism for removing a vehicle that is found to be malicious. A revocation list could be used but it would likely need to be distributed to all of the vehicles and could grow rather unwieldy.

A scheme using the Chinese Remainder Theorem (CRT) for group key distribution, and that perfectly preserves forward and backward secrecy by updating the group key every time the group membership changes, was proposed by Chauhan et al. [4]. While work was done to make the group key updates efficient to account for the high frequency of changes, the efficiency was achieved by using a single, symmetric key for all group members. While this also preserves the privacy of members within the group, it prevents the detection of Sybil attacks as well as the identification of malicious actors. In addition, vehicles must provide their real identities to the 2nd layer entities in order to join the system, which could result in abuse by malicious services.

The scheme proposed by Zhang & Wen [15], similarly to the scheme in [4], achieves high efficiency by forgoing group signatures. However, it suffers from the same problem of excessive key count as other schemes using 1:1 symmetric keys for communication. In this scheme, unlike other schemes discussed, the 1:1 keys are used only for communication between the vehicles and the RSU with no V2V communication considered. While this would result in fewer keys to maintain than systems requiring symmetric keys for all vehicle pairs, it is inefficient to require the vehicles to communicate via the RSUs, particularly in heavy traffic situations.

In [16], Xiong and Tang proposed a hierarchical cloud model for VANET with a decentralized top layer and two centralized layers for the RSUs and vehicles, respectively. In the scheme, nodes wishing to communicate exchange certificates obtained from a global authority. Nodes establish shared symmetric keys and use those for communication. While the exchange of ID certificates among peer nodes could allow the detection of Sybil attacks, it also allows the identification and tracking of vehicles, which violates driver privacy. Additionally, as discussed above, the use of 1:1 symmetric keys for group communication creates the need for each vehicle to retain and manage an impractical number of keys, as well as requiring each message to be separately encrypted for each node that should receive it.

### 3.3. Protocols Using Bilinear Pairing Based Cryptography

A scheme providing privacy protection when authenticating vehicles that join a VANET was proposed by Lu et al. [1]. The scheme includes checks for double-registration even when using encoded information instead of real identities to join the group. While this prevents a malicious user from obtaining two different pseudo-IDs for the same session, there is no procedure for later tracing the real identities of malicious nodes. Additionally, communication within the group uses a symmetric session key, which leaves no way to identify Sybil attacks using sender identity or unmask the sender of malicious messages.

The scheme by Alimohammadi and Pouyan [17] uses the privacy preserving authentication (PPA) scheme from [1] to sign messages, instead of only using it for authenticating initial membership requests as was done in the original scheme. The PPA scheme’s double-registration check is adopted for use in checking for two messages signed by the same vehicle without revealing the vehicle’s pseudo-ID. However, there is also no way to determine the pseudo-ID of the offending vehicle in order to remove it from the system. The test can only reveal that a malicious vehicle exists. In addition, since it is possible for members of the group to identify two or more messages were sent by the same vehicle, they can then track that vehicle.

Another scheme using bilinear pairings was proposed by Lim et al. [5]. This scheme used the short group signatures of Boneh, Boyen, Shacham (BBS). BBS signatures have a tracing key that can be used by the owner of that key to reveal message signer identities. However, the Lim et al. scheme contains no discussion of updating the group key values upon vehicle joins, departures, or even revocation. Instead, it proposes the creation of an efficient revocation list which is supposed to be used in validating future messages by comparing certificate information included with the messages to the certificate revocation list. Including this certificate information in messages would compromise vehicle privacy. In addition, the lack of group key updates endangers forward and backward secrecy.

### 3.4. Protocols Based on Smart Cards and Tamper-Proof Devices

De Sales et al. [18] present a scheme using a hierarchy of anonymity sets to create chains of increasingly specific identifiers. These sets are specified using certificates signed by a certificate authority. Tamper-proof devices prevent the certificates from being accessed in advance of their use. However, there is no mechanism to prevent attackers from either reusing old certificates or using the certificates provided in messages sent by other vehicles in order to create unique anonymity sets for use in a Sybil attack. In addition, this scheme requires the vehicles to detect the Sybil attacks. This places additional computational and storage burdens on the least well-equipped components of the system. Furthermore, each vehicle has a different set of vehicles within its communication range, which requires additional handling to reduce false positives.

### 3.5. Protocols Minimizing the Overhead Involved in Public Key Infrastructure

In [19], a scheme by Mansour et al. uses the CRT for efficient key updates and distribution. This scheme presents many of the same problems as the CRT-based scheme used in [4]. While the use of the CRT allows for the efficient update and re-distribution of the group key upon changes in membership, using a single, symmetric key to encrypt all group communications prevents the identification of malicious members.

### 3.6. Protocols That Use Cooperative Message Authentication or Batch Verification

A scheme combining BBS short group signatures with cooperative message authentication was proposed by Hao et al. [20]. However, the scheme provides no mechanisms for detecting Sybil attacks or updating the group keys to provide either forward or backward secrecy. In addition, RSUs are provided with the real identities and real private keys of the vehicles joining their groups, despite the fact that RSUs are exposed to the environment and susceptible to tampering.

Ali et al. [21] proposed to use signcryption in order to increase efficiency by combining signing and encryption into a single step. Additionally, the scheme allowed for batch unsigncryption. Unfortunately, the signcryption algorithm used required the public key of the message receiver, which prevents message broadcasting and would therefore require an impractical number of messages to be sent in order to relay information to a group of vehicles. In addition, the plaintext pseudo-IDs of the senders are sent along with the encrypted messages, which can result in privacy breaches as discussed above in Section 2.2. Privacy.

### 3.7. Summary

An ideal VANET should preserve the privacy of group members by hiding their real identities, prevent the tracking of individual vehicles by other vehicles, authenticate the members and their messages, allow the detection of malicious nodes attempting Sybil attacks, and provide methods for legal authorities to trace the identities of those nodes and remove their ability to access the system as well as preventing them from rejoining in the future. While the schemes discussed above all lack one or more of the desired features, our proposed scheme will unify all of them in a single system.

## 4. Proposed Scheme

This scheme proposes to overcome the shortcomings of the schemes discussed in Section 3. Table 1 shows the notations used in this paper.

### 4.1. System Model

**Figure 3:**
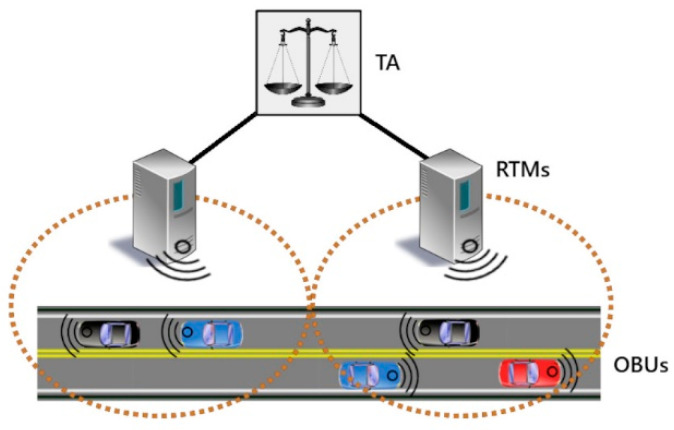
System diagram for proposed scheme.

We propose a hierarchical system for key management, with the level of trust decreasing with distance from the root node. Our proposed system, shown in Figure 3, can be used either with RSUs acting solely as communication relays or the cloud-based communication model shown in Section 1.

#### 4.1.1. Trusted Authority (TA)

At the top of the hierarchy sits the TA. The TA is a single, fully-trusted, highly-secure entity that is the ultimate authority within the system. It is charged with receiving and validating the real identities of the system entities through PKI certificates, generating and distributing group keys to each of the Regional Traffic Managers (RTMs) at the level below, and assigning OBUs to the appropriate RTMs based on physical locations. It connects to the RTMs via secure, hard-wired connections.

#### 4.1.2. Regional Traffic Managers (RTMs)

The level below the TA consists of a set of semi-trusted systems, called RTMs, that are in charge of monitoring OBUs for suspicious activity and reporting back to the TA. RTMs are assigned to geographic areas and may also manage regional services and provide information to the OBUs within their coverage area. They can communicate with the OBUs via either specialized road-side equipment known as RSUs, or via data connections over existing cellular data networks.

Because RTMs are only semi-trusted, they are not given access to the real identities of the OBUs in their regions in order to preserve user privacy. Instead, within their region each OBU is assigned a pseudo-ID. The pseudo-IDs can be used by an RTM to monitor the OBUs within its group in order to detect malicious activity and report those responsible to the TA, which can in turn reveal the real identities and take the appropriate actions.

#### 4.1.3. On-Board Units (OBUs)

OBUs are the lowest level of the system and are considered untrusted. They may attempt to interfere with other OBUs as well as manipulate the system for their own advantage. OBUs must obtain PKI certificates in order to be validated by the TA and join an RTM group, but in a real-world system it will of course be possible for malicious actors to obtain a valid ID certificates through various means. Therefore, it must be assumed that malicious actors are inside the system attempting to cause harm. In order to prevent this, the RTMs monitor OBU messages for irregularities and OBU identities are concealed from the other OBUs.

### 4.2. Preliminary Definitions

$G_{1}$ and $G_{2}$ are two bilinear, multiplicative, cyclic groups of prime order p. $g_{1}$ is a generator of $G_{1}$ and $g_{2}$ is a generator of $G_{2}$. ψ is a computable isomorphism from $G_{2}$ to $G_{1}$, with $\psi \left(g_{2}\right)=g_{1}$. e is a computable map $e:G_{1}\times G_{2}\to G_{T}$ and $e\left(g_{1},g_{2}\right)\neq 1$.

### 4.3. Process

**Figure 4:**
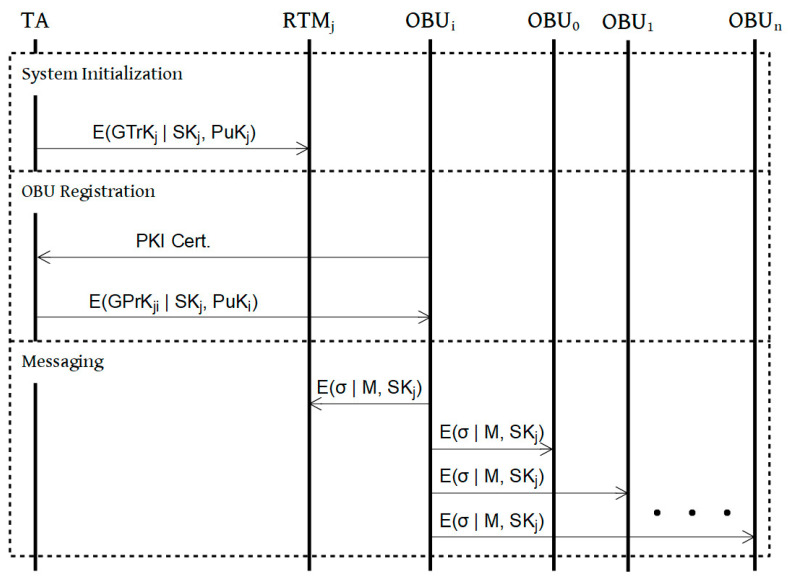
Process.

This scheme follows the BBS short group signature scheme presented in [22] and consists of 5 primary parts: initialization, registration, messaging, tracing, and revocation, which are presented in the following sections. Figure 4 shows the steps of the process.

#### 4.3.1. System Initialization

Prior to system operation, the TA generates the initial batch of group keys for each RTM in the system as detailed below. The keys will be distributed to the RTMs via a secure channel. When a group key update event occurs for an RTM, new group private and tracing keys for that RTM will be generated using the same method.

Step 1: For each RTM index j, TA generates the group keys. First, it randomly selects $h_{j}\leftarrow G_{1}\backslash \left\{1_{G_{1}}\right\}$ and $\zeta_{1j},\;\zeta_{2j}\leftarrow {\mathbb{Z}}_{p}^{*}$ and sets $u_{j},\;v_{j}\in G_{1}$ such that $u_{j}^{\zeta_{1j}}=v_{j}^{\zeta_{2j}}=h_{j}$. Then it randomly selects the group master key $\gamma_{j}\leftarrow {\mathbb{Z}}_{p}^{*}$ and sets $w_{j}=g_{2}^{\gamma_{j}}$. The group master key will be saved for use later making the group private keys for the OBUs. The group public key, GPuK_j_, is now the tuple $\left(g_{1},\;g_{2},\;h_{j},\;u_{j},\;v_{j},\;w_{j}\right)$. The group tracing key, GTrK_j_, is $\left(\zeta_{1j},\;\zeta_{2j}\right)$.
Step 2: The TA will also generate a symmetric key to be used by a members of group j for encrypting messages within the group. The shared symmetric key, SK_j_, is randomly chosen.
Step 3: The tracing and symmetric keys for each group j will be sent to RTM_j_.

The TA retains the group master key and transmits the group tracing and symmetric keys to the RTM encrypted by the RTM’s public key. The RTM has no knowledge of the group master key. The group tracing key can only be used to reveal pseudo-IDs. The real IDs of all OBUs in the group will be protected even from the managing RTM.

#### 4.3.2. OBU Registration

Because the TA possesses the group master keys, OBUs need only contact the TA in order to both validate their PKI certificates and obtain their group private key. The RTMs are not involved in the process of OBU registration. The TA will determine which RTM an OBU should be assigned to using either location data from RSUs—in the RSU-based system model—or OBU GPS data—in a cloud-based system model.

Step 1: When OBU_i_ enters RTM_j_’s area it sends its public key certificate to the TA so that the TA can validate OBU_i_ and provide it with group keys and pseudo-ID.
Step 2: The TA calculates the private key for OBU_i_ within RTM_j_’s group. OBU_i_’s group private key, GPrK_ji_, will be computed by the TA using the group master key $\gamma_{j}.$ In order to do this, the TA randomly selects $x_{ji}\leftarrow {\mathbb{Z}}_{p}^{*}$ then it computes $A_{ji}\leftarrow g_{1}^{1/\left(\gamma_{j}+x_{ji}\right)}$.
Step 3: The TA sets GPrK_ji_ to be the tuple $\left(A_{ji},x_{ji}\right)$ and sends it to OBU_i_ encrypted with OBU_i_’s public key. RTM_j_ has no knowledge of GPrK_ji_.

The first element of the private key tuple, $A_{ji}$, is the pseudo-ID of OBU_i_. Although pseudo-IDs will update when a group key recalculation is triggered, this scheme also ensures that no OBU will have the same pseudo-ID across multiple RTMs, therefore reducing the chance of tracking the OBU by malicious actors. The TA saves a mapping of the pseudo-ID to the real identity of OBU_i_. This mapping can be used to recover the real identity of an OBU for law enforcement or billing purposes.

#### 4.3.3. Messaging

OBUs sign all their messages to other OBUs and the RTM using privacy-preserving group signatures for verification. Messages are encrypted using a group symmetric key and broadcast to all group members. The signatures are constructed as detailed below:

Step 1: First, OBU_i_ chooses two random values $\alpha ,\;\beta \leftarrow {\mathbb{Z}}_{p}$. Different random values should be chosen each time a message is signed so the other group members cannot link which messages were signed by the same vehicle, which could allow malicious nodes to track other group members.
Step 2: Using the random numbers selected above, the group public key, and OBU_i_’s group private key, OBU_i_ computes:
  $$T_{1}\leftarrow u_{j}^{\alpha },\;T_{2}\leftarrow v_{j}^{\beta },\;\mathrm{and}\;T_{3}\leftarrow A_{ji}h_{j}^{\alpha +\beta }\;\mathrm{as}\;\mathrm{well}\;\mathrm{as}\;\delta_{1}\leftarrow x_{ji}\alpha \;\mathrm{and}\;\delta_{2}\leftarrow x_{ji}\beta$$

The OBUs include a challenge value with their signatures so receivers can validate the message contents and verify the sender is a group member without knowing the pseudo-ID of the sender. This preserves the privacy of the OBUs and prevents tracking within the group.

Step 3: OBU_i_ constructs the challenge. It selects random numbers $r_{\alpha },\;r_{\beta },\;r_{x},\;r_{\delta_{1}},\;r_{\delta_{2}}\leftarrow {\mathbb{Z}}_{p}$ and calculates
  $$R_{1}\leftarrow u_{j}^{r_{\alpha }},\;R_{2}\leftarrow v_{j}^{r_{\beta }},\;R_{3}\leftarrow e{\left(T_{3},\;g_{2}\right)}^{r_{x}}\cdot e{\left(h_{j},w_{j}\right)}^{-r_{\alpha }-r_{\beta }}\cdot e{\left(h_{j},\;g_{2}\right)}^{-r_{\delta_{1}}-r_{\delta_{2}}},\;R_{4}\leftarrow T_{1}^{r_{x}}\cdot u_{j}^{-r_{\delta_{1}}},\;\mathrm{and}\;R_{5}\leftarrow T_{2}^{r_{x}}\cdot v_{j}^{-r_{\delta_{2}}}$$

The challenge value is constructed using a public hash function, the message, and the newly computed values as shown:

$$c\leftarrow H\left(M,\;T_{1},\;T_{2},T_{3},R_{1},R_{2},\;R_{3},\;R_{4},\;R_{5}\right)\in {\mathbb{Z}}_{p}$$

The signature consists of the challenge as well as the values required for the receivers to recreate the challenge using the group public key and confirm the signature is valid. None of the values reveal enough information for the receivers to recover the pseudo-ID of the sender or otherwise link messages sent by the same OBU provided values for α and β are re-chosen for every signature as described in Step 1. If α and β values are reused, the $T_{1}$, $T_{2}$, and $T_{3}$ components of the signature will remain the same across different messages, which would allow any receiver to link messages sent by the same OBU without knowing any secret information.

Step 4: In order to make the signature, OBU_i_ computes:
  $$s_{\alpha }=r_{\alpha }+c\alpha ,\;s_{\beta }=r_{\beta }+c\beta ,\;s_{x}=r_{x}+cx_{ji},\;s_{\delta_{1}}=r_{\delta_{1}}+c\delta_{1},\;\mathrm{and}\;s_{\delta_{2}}=r_{\delta_{2}}+c\delta_{2}.$$

The signature is then $\sigma \leftarrow \left(T_{1},T_{2},T_{3},c,s_{\alpha },s_{\beta },s_{x},s_{\delta_{1}},s_{\delta_{2}}\right)$. Receivers can verify the signature and message integrity without knowing the signer identity by recreating the challenge value using the group public key and the values included in the signature.

Step 5: Receivers use the signature and group public key to compute
  $${\tilde{R}}_{1}\leftarrow u_{j}^{s_{\alpha }}/T_{1}^{c},\;{\tilde{R}}_{2}\leftarrow v_{j}^{s_{\beta }}/T_{2}^{c},\;{\tilde{R}}_{3}\leftarrow e{\left(T_{3},g_{2}\right)}^{s_{x}}\cdot e{\left(h_{j},w_{j}\right)}^{-s_{\alpha }-s_{\beta }}\cdot e{\left(h_{j},g_{2}\right)}^{-s_{\delta_{1}}-s_{\delta_{2}}}\cdot {\left(\frac{e\left(T_{3},w_{j}\right)}{e\left(g_{1},g_{2}\right)}\right)}^{c},\;{\tilde{R}}_{4}\leftarrow T_{1}^{s_{x}}/u_{j}^{s_{\delta_{1}}},\;\mathrm{and}\;{\tilde{R}}_{5}\leftarrow T_{2}^{s_{x}}/v_{j}^{s_{\delta_{2}}}.$$

Receivers can then check the challenge value by testing.

$$c=H\left(M,T_{1},T_{2},T_{3},{\tilde{R}}_{1},{\tilde{R}}_{2},{\tilde{R}}_{3},{\tilde{R}}_{4},{\tilde{R}}_{5}\right)$$

At no point do receivers know the pseudo-ID of the OBU that signed the given message.

#### 4.3.4. Pseudo-ID Tracing

RTMs monitor group messages and perform tracing to reveal the pseudo-IDs of message senders. The tracing is required to perform Sybil attack detection, but it occurs with neither compromising the real identity of the sender nor revealing the pseudo-ID of the message senders to the other OBUs, which are less trusted than the RTMs.

Using the group tracing key and values in the signature, RTM_j_ can reveal the pseudo-ID of the OBU that signed each message using the simple equation:

$$A_{ji}\leftarrow T_{3}/\left(T_{1}^{\zeta_{1j}}\cdot T_{2}^{\zeta_{2j}}\right).$$

After the pseudo-ID of a message sender is obtained, the RTM can evaluate the message contents in order to detect Sybil attacks as well as other malicious behavior. RTMs can store pseudo-IDs and message history in order to aid in the detection of malicious or malfunctioning nodes. The detection of malicious OBUs by RTMs is a data analysis problem and outside of the scope of this paper, but some possible examples of how data analysis could be used to detect Sybil attacks include: checking the message frequency for OBUs sending messages more often than they should, sanity checking location messages to detect OBUs reporting physically impossible movements, checking status messages for OBUs reporting conflicting or otherwise inconsistent status information, etc.

#### 4.3.5. Revocation and Group Key Updates

There are two cases in which the group keys must be revoked and regenerated. The first case is when a malicious node is detected and removed from the group. The second case is when members leave the group voluntarily by exiting the area or quitting the service. In either case, when a group key update is required, the TA will generate a new master and associated keys, and transmit the appropriate keys to the RTM as detailed in Section 4.3.1. System Initialization. The TA will then generate new group keys for all of the OBUs in the group and distribute them as described in Section 4.3.2. OBU Registration.

*Case 1*: Revocation of Malicious OBUs

When a malicious OBU is detected by an RTM, the offending OBU should be removed from the system so that it may no longer inject malicious messages. In order to avoid maintaining a potentially long and cumbersome revocation list, the proposed scheme will revoke and reissue the group keys for all other OBUs when a malicious OBU is detected.

The reissuance of new group keys will begin when the RTM notifies the TA of a malicious OBU. The TA will randomly choose a new group master key, then calculate the corresponding group public key and group tracing key, and transmit the new group tracing key to the RTM. After that, for each pseudo-ID in the group other than the revoked OBU, the TA will generate a new group private key and securely distribute those keys to the corresponding OBUs.

The real ID of the malicious OBU will be reported by the TA to the relevant authorities and placed in a TA-local revocation list to prevent re-registration.

*Case 2*: Revocation of Honest OBUs

Unlike the case of malicious OBUs, there is no need to immediately revoke an OBU that leaves the group under normal circumstances, e.g., due to exiting the area of RTM coverage. In fact, attempting to revoke every OBU immediately upon leaving can lead to an infeasible amount of key update calculations and messages. This can be shown using the following formulas:

*vehicles per second = vehicle interval/vehicle speed* required key update rate = vehicles per second * number of lanes

**Figure 5:**
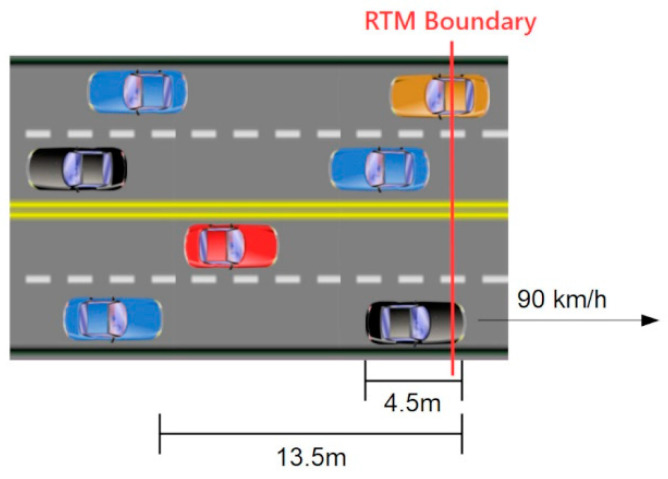
Traffic diagram.

Consider a relatively busy city highway with traffic moving at 90 km per hour or 25 m per second. Assume an average vehicle length of 4.5 m and a separation of two vehicle lengths for 13.5 m total per vehicle interval, including the vehicle’s own length and the buffer. (Shown in Figure 5) Under those conditions, even if we only consider a single lane of traffic, at that moderate speed one vehicle will leave the group every 0.54 s. If the traffic travels in two lanes in both directions, for a total of four lanes, one vehicle will cross an RTM boundary every 0.135 s.

**Figure 6:**
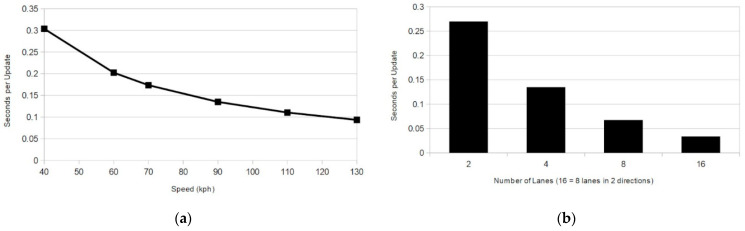
Seconds between key updates due to departing OBUs: (**a**) Single lane of traffic at various speeds; (**b**) Traffic moving at 90 kph.

As shown in Figure 6, the frequency of updates required to maintain perfect backward secrecy every time an OBU leaves the group can become impractical for high traffic areas without even considering traffic on all roads that might cross an RTM boundary. In Figure 6b, it can be seen in the case of traffic moving at 90 kph on a four lane highway that a vehicle will leave the group every 135 ms.

Standard J2735, the Message Set Dictionary for the Dedicated Short Range Communication (DSRC) standard, suggests vehicles should broadcast the Basic Safety Message (BSM) approximately every 100 ms [23]. Even in the rather generous scenario considered in the previous paragraph, the group key would be used by each vehicle for only a single safety message on average. The use of a revocation list could prolong the life of the group key, but the list will quickly grow large. This makes it inefficient to store, process, and, importantly, transmit to new group members.

Instead of attempting to perfectly maintain backward secrecy, the proposed scheme uses an interval of validity to periodically force the revocation of group keys at some pre-determined time interval. When the time interval is exceeded, the TA will begin the process of revoking and reissuing the group keys as described above for revocation of malicious OBUs.

## 5. Analysis

We will next examine the proposed scheme with respect to the security concerns outlined in Section 2: VANET Security Concerns.

### 5.1. Authentication

In the proposed scheme, the TA performs the authentication of an OBU’s real identity via PKI certificate before issuing group keys when an OBU sends a request to join an RTM coverage area. Requiring proof of real identity can reduce the ability of malicious OBUs to enter the group as they must first pass an offline identity verification process in order to obtain PKI certificates validating their real identities.

### 5.2. Privacy

The proposed scheme protects driver privacy in several ways. First, the real identity of the OBUs are known only to the TA. In the event that other OBUs, or even RTMs, are compromised those entities cannot obtain the real identity of other OBUs from the group signature as no information from the real identity is used in the generation of either the signature or the OBU’s group private key.

In addition, the pseudo-IDs of the OBUs are protected from each other by the privacy preserving short group signatures, making it difficult for an OBU to associate messages with their senders. Recall the value $A_{ji}$ is the pseudo-ID for OBU i in RTM group j. $A_{ji}$ is included in the signature during the calculation of $T_{3}\leftarrow A_{ji}h_{j}^{\alpha +\beta }$. However, in order to obtain $A_{ji}$ given $T_{1}$, $T_{2}$, and $T_{3}$ an attacker would have to solve the decision linear problem. That is, given $u_{j}$, $v_{j}$, $h_{j}$, $u_{j}^{\alpha }$ (from $T_{1}$), $v_{j}^{\beta }\;$(from $T_{2}$) determine the value for $h_{j}^{\alpha +\beta }$ distinct from any other random member of group $G_{1}$ [22]. Because, among members of a given group, only the RTM possess the tracking key for that group, only the RTM can obtain the value of $A_{ji}$ from a signature easily.

Concealing the pseudo-IDs from all but the RTMs helps to protect the privacy of OBUs by hindering both the monitoring of a specific OBU within the group as well as the creation of a message history associated with a single OBU. The latter is important because, as previously noted, such information could be reverse engineered to infer the real identities of drivers from even anonymized location data sets.

### 5.3. Tracing

Although the proposed scheme provides real identity authentication before an OBU can join a group, it is still possible that a malicious OBU may obtain valid proof of real identity by methods such as successful forgery of required offline proof of identity documentation, theft of certified identity data from a legitimate user, or even by simply providing its actual real identity. In the case that a malicious OBU is able to join the system, an RTM can use the group tracing key to detect and reveal the pseudo-ID of the offending OBU. The proof of $A_{ji}\leftarrow T_{3}/\left(T_{1}^{\zeta_{1j}}\cdot T_{2}^{\zeta_{2j}}\right)$ can be seen simply from the use of the tracing key values $\left(\zeta_{1j},\;\zeta_{2j}\right)$ to generate $h_{j}$ from $u_{j}$ and $v_{j}$. Because $u_{j}^{\zeta_{1j}}=v_{j}^{\zeta_{2j}}=h_{j}$, simple substitution into the formula for $A_{ji}$ above shows:

$$A_{ji}\leftarrow \frac{T_{3}}{T_{1}^{\zeta_{1j}}\cdot T_{2}^{\zeta_{2j}}}$$

$$\frac{A_{ji}h_{j}^{\alpha +\beta }}{u_{j}^{\alpha \zeta_{1j}}\cdot v_{j}^{\beta \zeta_{2j}}}$$

$$\frac{A_{ji}h_{j}^{\alpha +\beta }}{u_{j}^{\zeta_{1j}\alpha }\cdot v_{j}^{\zeta_{2j}\beta }}$$

$$\frac{A_{ji}h_{j}^{\alpha +\beta }}{h_{j}^{\alpha }\cdot h_{j}^{\beta }}$$

$$\frac{A_{ji}h_{j}^{\alpha +\beta }}{h_{j}^{\alpha +\beta }}$$

Once the pseudo-ID is revealed, it can be passed to the TA which can map it to the real identity associated with the OBU and take appropriate action including regenerating group keys to exclude the malicious OBU, preventing the attacker from re-joining the system using the same real identity, and reporting the real identity to external authorities.

### 5.4. Sybil Resistance

The proposed scheme allows for the detection of Sybil attacks by providing RTMs with the ability to reveal the pseudo-IDs of message signers and thereby detect OBUs that are attempting to send messages appearing to come from multiple OBUs. In addition, while one part of the group private key tuple, $x_{ji}$, is chosen randomly, a valid message signature requires a pseudo-ID value generated using the master key. As only the TA knows the master key, it is impossible for an OBU, or any combination of colluding OBUs, to forge multiple pseudo-IDs that are capable of generating valid signatures. Additionally, the master key cannot be obtained from $w_{j}=g_{2}^{\gamma_{j}}$ without solving the discrete logarithm problem.

Finally, without the tracing key, which is held only by the group RTM, an OBU cannot determine the pseudo-IDs of other OBUs (as discussed above in Section 5.2. Privacy) and is therefore prevented from masquerading as other OBUs in the group in order to perform a Sybil attack in that way.

### 5.5. Backward Secrecy & Forward Secrecy

Due to the high rate of change of group composition in VANETs, an excessive number of key updates can be required to change the keys every time an OBU joins or leaves the group. By periodically updating group keys at a predetermined rate, the proposed scheme maintains sufficient levels of backward and forward secrecy without recalculating and redistributing the group keys excessively. The appropriate rate of group key updates can be adjusted following an analysis of traffic patterns as well as taking into consideration the available network capacity and throughput of the hardware used for implementation of the VANET.

As for the consequences to security, in the case of backward secrecy, members who leave the group in a normal manner, e.g., simply leave the coverage area and are not removed for malicious behavior, can be assumed to be sufficiently trustworthy to keep access to the group keys until the next scheduled update interval. In the case of forward secrecy, the dynamic nature of VANETs means that there is limited value to accessing old messages. There is no need to maintain complete forward secrecy as by the time old data is obtained, it is likely no longer useful.

### 5.6. Revocation

In the proposed scheme, both the pseudo-IDs and real identities of malicious OBUs can be obtained promptly by communication between the RTMs and TA, and such users can be both removed from the group and subsequently prevented from rejoining. Instead of maintaining and distributing an often cumbersome revocation list, the group keys will be recalculated and redistributed immediately. Only the TA will retain a list of the real identities of revoked users in order to prevent them from joining any groups at any future time.

Using the revocation list of real identities will, of course, prevent malicious users from rejoining the group in order to cause more mischief, but it will also prevent an attacker from attempting a denial-of-service (DOS) attack by constantly rejoining and immediately causing trouble in order to trigger excessive cycles of revocation and reissuance of all group keys. In order to attempt such a DOS attack, the attacker would have to obtain a large number of valid PKI certificates for different falsified or stolen identities. The high cost of amassing these faked identities, which require a process of offline verification by outside authorities, should make such attacks infeasible.

### 5.7. Prevention of Other Insider Attacks

As a consequence of providing the ability to link message contents to their senders, the proposed scheme also enables the detection of a range of other insider attacks. By examining message contents in light of sender identity, it is possible to detect activities that either violate the system spec, such as a vehicle that sends updates at greater than the specified rate, or are physically impossible, such as a vehicle that reports consecutive positions exceeding maximum vehicle speeds. The list of potentially detectable attacks includes: broadcast tampering, spamming/DOS, node impersonation, position faking, and illusion attacks [24].

Although not frequently discussed in VANET security literature, our scheme’s ability to reveal the true identity of a vehicle can help in the area of role-based access control (RBAC), which may become a hot topic for VANET in the near future. RBAC has been traditionally used in the context of organizational security. As the name implies, it seeks to control system access by assigning users to a set of roles where each role has a specific set of permissions defined by security administrators [25]. RBAC can be expanded to control access to databases using context-sensitive information, such as location at time of request [26].

In a VANET, a vehicle could be given limited access to data based on location and/or vehicle type (civilian, police, ambulance, construction, transit, etc.). Our scheme would allow access controllers to trace the identity of a vehicle claiming a privileged role for confirmation. Because our scheme allows tracing at the RTM level, it would be particularly helpful in the case of fog-based access control, which is an access control scheme where processing is offloaded to intermediate nodes of an IoT system [27].

### 5.8. Comparison to Existing Schemes

As shown in Table 2, the proposed scheme provides all of the functionality required to detect Sybil attacks in a VANET while simultaneously preserving vehicle privacy. In contrast, none of the existing schemes examined met all of the specified requirements. We believe our scheme provides an important step in filling that functionality gap.

## 6. Conclusions

In this paper we proposed a scheme for detecting Sybil attacks in VANETs while also preserving vehicle privacy and message confidentiality. In our scheme a fully-trusted TA is the only entity aware of the real identities of other members of the system. RTMs and OBUs, which are exposed to the environment and therefore at greater risk of compromise, are unable to either track vehicles across the system or to reveal the locations of owners based on vehicle registration information. The TA generates and distributes group keys to the RTMs and OBUs directly to ensure this privacy. Additionally, OBUs must register with the TA using PKI certificates in order to obtain the group keys, which provides a first step of validation and also allows the reporting of OBUs that are later found to be malicious.

After the group keys are received, the OBUs communicate using messages signed with short group signatures. The signatures validate that messages received come from an authenticated group member, as well as allowing RTMs to obtain the pseudo-ID information for the message senders. Once obtained, the pseudo-IDs can be used along with the message contents to detect attempted Sybil attacks. RTMs can report the pseudo-IDs of malicious nodes to the TA, which can obtain the real identities. The pseudo-IDs are unique for each RTM coverage area so that malicious RTMs cannot use the pseudo-ID information to track vehicles after they leave its own area of coverage. In addition, OBUs cannot obtain the pseudo-IDs of other OBUs from their signatures, so OBUs cannot track other OBUs in the group by their signatures. Finally, we provided support for the argument that maintaining complete forward and backward secrecy is impractical in a highly dynamic environment such as VANET due to the frequency of group composition change in real-world situations.

In future work we intend to investigate the performance and network usage of the proposed scheme and identify ways in which the scheme’s efficiency can be improved. We also plan to examine how the concept of authorization could be applied in VANET, for example limiting some data access to certain vehicles such as ambulances or police cars, while still maintaining privacy.

## Figures and Tables

**Table 1:** Notations.

Symbol	Definition
i	Index of OBU
j	Index of Regional Traffic Manager (RTM)
PuK_i_	Public key for OBU index i, obtained from PKI certificate
PuK_j_	Public key for RTM index j, obtained prior to initialization
A_ji_	Pseudo-ID of OBU index i in the coverage area of RTM index j
ɣ_j_	Master key for RTM index j
GPuK_j_	Group public key for RTM index j
GTrK_j_	Group tracing key for RTM index j
GPrK_ji_	Private key of OBU index i within the group for RTM index j
SK_j_	Symmetric key for all members of the group for RTM index j
c	Message challenge value
σ	Message signature

**Table 2:** Comparison of proposed scheme to existing group schemes. Y = provided, / = partially provided, N/A = not applicable.

Feature	[20]	[1]	[17]	[4]	[14]	[5]	[19]	Proposed
Forward Secrecy	No	/(Timed)	/(Once per Day)	**Y (Always update)**	No	No	**Y (Always update)**	/(Timed)
Backward Secrecy	No	**Y (Leave updates)**	/(Once per Day)	**Y (Always update)**	No	No	**Y (Always update)**	/(Timed & revoked)
Sybil Resistance	No	**Y (Unique signature)**	**Y (Signatr. matching)**	No	**Y (1:1 sym. keys)**	No	No	**Y (RTM checking)**
Real ID Exposure Level	RSU	**TA**	RSU	KMS	**TA**	L-RSU	No ID Check	**TA**
Tracking Prevention	**Y (Group signature)**	**Y (Group key)**	No	**Y (Group key)**	No	**Y (Group signature)**	**Y (Group key)**	**Y (Group signature)**
Message Authentication	**Y (Group signature)**	**Y (Group key)**	**Y (Group signature)**	**Y (Group key)**	**Y (Group signature)**	**Y (Group signature)**	**Y (Group key)**	**Y (Group signature)**
Tracing	**Y (Private key)**	No	No	No	No	**Y (Private key)**	No	**Y (Private key)**
Member Revocation	**Y (RSU)**	**Y (RSU)**	No	No	No	/(Can’t join new)	No	**Y (TA)**

## References

[1] Lu R., Lin X., Liang X., Shen X. (2012). A Dynamic Privacy-Preserving Key Management Scheme for Location-Based Services in VANETs. IEEE Trans. Intell. Transp. Syst..

[2] Vishwakarma R., Barskar R., Ahirwar M. Secure Key Management in Vehicular Ad-Hoc Network: A Review. Proceedings of the 2016 International Conference on Signal Processing, Communication, Power and Embedded System (SCOPES).

[3] Sheikh M.S., Liang J., Wang W. (2019). A Survey of Security Services, Attacks, and Applications for Vehicular Ad Hoc Networks (VANETs). Sensors.

[4] Chauhan K.K., Kumar S., Kumar S. The Design of a Secure Key Management System in Vehicular Ad hoc Networks. Proceedings of the 2017 Conference on Information and Communication Technology (CICT’17).

[5] Lim K., Liu W., Wang X., Joung J. (2019). SSKM: Scalable and Secure Key Management Scheme for Group Signature Based Authentication and CRL in VANET. Electronics.

[6] Petit J., Dietzel S., Kargl F., Gkoulalas-Divanis A., Bettini C. (2018). Privacy of Connected Vehicles. Handbook of Mobile Data Privacy.

[7] Zhou T., Choudhury R.R., Ning P., Chakrabarty K., Cao G., Kravets R. (2007). Privacy-Preserving Detection of Sybil Attacks in Vehicular Ad Hoc Networks. Proceedings of the 4th International ICST Conference on Mobile and Ubiquitous Systems: Computing, Networking and Services (MobiQuitous).

[8] Parno B., Perrig A. Challenges in Securing Vehicular Networks. Proceedings of the Fourth Workshop on Hot Topics in Networks (HotNets-IV).

[9] Raya M., Hubaux J.-P. (2007). Securing vehicular ad hoc networks. J. Comput. Secur..

[10] Mejri M.N., Ben-Othman J., Hamdi M. (2014). Survey on VANET security challenges and possible cryptographic solutions. Veh. Commun..

[11] Douriez M., Doraiswamy H., Freire J., Silva C.T. Anonymizing NYC Taxi Data: Does It Matter?. Proceedings of the 3rd IEEE International Conference on Data Science and Advanced Analytics (DSAA 2016).

[12] Alzaid H., Park D., Nieto J.G., Boyd C., Foo E., Hailes S., Sicari S., Roussos G. (2010). A Forward and Backward Secure Key Management in Wireless Sensor Networks for PCS/SCADA. Lecture Notes of the Institute for Computer Sciences, Social Informatics and Telecommunications Engineering, Proceedings of the International Conference on Sensor Systems and Software (S-CUBE 2009), Pisa, Italy, 7–9 September 2009.

[13] Manivannan D., Moni S.S., Zeadally S. (2020). Secure authentication and privacy-preserving techniques in Vehicular Ad-hoc NETworks (VANETs). Veh. Commun..

[14] Gao T., Qi J., Barolli L., Leu F.Y., Enokido T., Chen H.C. (2019). An Anonymous Access Authentication Scheme for VANETs Based on ID-Based Group Signature. Lecture Notes on Data Engineering and Communications Technologies, Proceedings of the 13th International Conference on Broadband and Wireless Computing, Communication and Applications (BWCCA 2018), Taichung, Taiwan, 27–29 October 2018.

[15] Zhang Y., Wen F., Bian F., Liu X., Hsu H.-M. (2019). A Lightweight Secure and Efficient Authentication and Key Agreement Protocol for VANET. IOP Conference Series: Earth and Environmental Science, Proceedings of the 6th Annual 2018 International Conference on Geo-Spatial Knowledge and Intelligence (GSKI 2018), Wuhan, Hubei, China, 14–16 December 2018.

[16] Xiong W., Tang B., Yuan H., Geng J., Liu C., Bian F., Surapunt T. (2018). A Cloud Based Three Layer Key Management Scheme for VANET. Communications in Computer and Information Science, Proceedings of the 5th International Conference of Geo-Spatial Knowledge and Intelligence (GSKI 2017), Chiang Mai, Thailand, 8–10 December 2017.

[17] Alimohammadi M., Pouyan A.A. Sybil Attack Detection Using a Low Cost Short Group Signature in VANET. Proceedings of the 12th International Iranian Society of Cryptology Conference on Information Security and Cryptology (ISCISC 2015).

[18] De Sales T.B.M., Perkusich A., De Sales L.M., De Almeida H.O., Soares G., De Sales M. (2016). ASAP-V: A privacy-preserving authentication and sybil detection protocol for VANETs. Inf. Sci..

[19] Mansour A., Malik K.M., Alkaff A., Kanaan H. (2020). ALMS: Asymmetric Lightweight Centralized Group Key Management Protocol for VANETs. IEEE Trans. Intell. Transp. Syst..

[20] Hao Y., Cheng Y., Zhou C., Song W. (2011). A Distributed Key Management Framework with Cooperative Message Authentication in VANETs. IEEE J. Sel. Areas Commun..

[21] Ali I., Lawrence T., Omala A.A., Li F. (2020). An Efficient Hybrid Signcryption Scheme with Conditional Privacy-Preservation for Heterogeneous Vehicular Communication in VANETs. IEEE Trans. Veh. Technol..

[22] Boneh D., Boyen X., Shacham H. Short Group Signatures. Proceedings of the 24th Annual International Cryptology Conference (CRYPTO 2004).

[23] Cronin B. Vehicle Based Data and Availability. https://www.its.dot.gov/itspac/october2012/PDF/data_availability.pdf.

[24] Afzal H., Kumar M. (2020). Security of Vehicular Ad-Hoc Networks (VANET): A survey. Journal of Physics: Conference Series, Proceedings of the 3rd National Conference on Computational Intelligence (NCCI 2019), Bangalore, Karnataka, India, 5–6 December 2019.

[25] Sandhu R.S., Coyne E.J., Feinstein H.L., Youman C.E. (1996). Role-Based Access Control Models. IEEE Comput..

[26] Kayes A.S.M., Rahayu W., Dillon T., Chang E., O’Conner L. (2018). Accessing Data from Multiple Sources Through Context-Aware Access Control. Proceedings of the 17th IEEE International Conference on Trust, Security and Privacy in Computing and Communications (IEEE TrustCom 2018).

[27] Kayes A.S.M., Rahayu W., Watters P., Alazab M., Dillon T., Chang E. (2020). Achieving security scalability and flexibility using Fog-Based Context-Aware Access Control. Future Gener. Comput. Syst..

